# Comparison of allelopathic effects of two typical invasive plants: *Mikania micrantha* and *Ipomoea cairica* in Hainan island

**DOI:** 10.1038/s41598-020-68234-5

**Published:** 2020-07-09

**Authors:** Huiyan Ma, Yu Chen, Jinhui Chen, Yunqiu Zhang, Tian Zhang, He He

**Affiliations:** 1 College of Forestry, Hainan University, Haikou, 570100 Hainan China; 2 College of Ecology and Environment, Hainan University, Haikou, 570100 Hainan China

**Keywords:** Biochemistry, Plant sciences

## Abstract

*Mikania micrantha* and *Ipomoea cairica* are two invasive plants widely distribute and seriously damage in Hainan island. In this study, the leaves extracts of two weeds were collected and determined for their allelopathic potentials on *Chrysanthemum coronarium*. The phytotoxicity bioassay showed that when the extract concentration was 50 and 100 mg/ml, the inhibited effects of *M. micrantha* on growth of *C. coronarium* were greater than by *I. cairica*. However, when the extract concertation at 400 mg/ml, the opposite inhibited effects were observed. We speculated this phenomenon was caused by different allelopathic compounds. Therefore, using gas chromatography–mass spectrometry, 19 and 23 compounds were identified respectively, benzoic acid and cinnamic acid were the main components in the two leaves extracts, which were selected to carry out the further bioassays. Subsequent bioassay results showed the effects of two allelochemicals on morphological index and chlorophyll content and POD activity were all negative to *C. coronarium*, whereas the content of MDA and activity of SOD, CAT represented adverse changes. Moreover, the inhibitions by cinnamic acid were generally greater than those by benzoic acid. Thus, the phenolic acids played the most crucial roles in the allelopathic effccts of *M. micrantha* and *I. cairica* leaves extracts.

## Introduction

Exotic plant invasion is an important part of global change, and one of the most severe environmental problems facing humanity^1–3^. Hainan island, a tropical province of China, with light, temperature, water and soil conditions suitable for plant propagation and growth. However, its ecological environment is relatively fragile. Coupled with the increasing frequency of foreign trade and exchanges in recent years, a large number of exotic plants have settled down. *Mikania micrantha* is one of the most harmful invasive alien weeds, native to South and Central America, and known as mile-a-minute weeds^4–6^, while *Ipomoea cairica* is the second most important invasive plant in Hainan island in recent years^7^. They are perennial twining lianas with similar ecological niches, which can be associated with each other to form dominant populations in new habitats, seriously affecting agricultural and forestry production and destroying biodiversity^8,9^.

To clarify the invasion mechanism of *M. micrantha* and *I. cairica*, scholars have done a lot of research. On the one hand, some studies attributed their successful invasion to substantial reproduction and wide eco-physiological tolerance, which can efficiently capture and utilize environmental resources^10,11^. *I. cairica* not only has a higher photosynthetic rate and utilization rate of photosynthetic nitrogen, but also has a higher specific leaf area and lower leaf construction cost, which reflects its advantages in the utilization efficiency of carbon and nitrogen resources^12^. Similarly, the leaves of *M. micrantha* also have a relatively higher photosynthetic capacity and water using efficiency^13,14^. On the other hand, the allelopathic effects of *M. micrantha* and *I. cairica* are widely received as an important role in biological invasion. Allelopathy is defined as the direct or indirect inhibitory or stimulatory effects of one plant on another (including microorganisms) by producing compounds that escape from plants into the environment, and is concerned to be a powerful chemical weapon during the invasion process of alien plants^15–17^. It was found the extracts of *M. micrantha* and *I. cairica* can effectively inhibit the germination and seedling growth of tested plants^18–21^. Moreover, the soil treated with a higher concentration of *M. micrantha* leaves extracts was also reported to actively suppress seedling growth^22^. Previous studies have shown that both weeds have allelopathic effects, however, little knowledge has been reported about their effects on the same species. Whether *C. chinensis* will produce a stronger allelopathic effect against the invasion of *M. micrantha* is unknown. If so, is the growth inhibited effect of the two weeds on the colony and nearby grassland species caused by the different allelochemicals produced by the two? As early as 1964, scholars such as Muller suggested that there were intimate relationships between chemicals extracted from allelopathic plants and its distinguished competition in the new ranges^23^. And this competitiveness varied with the composition of the compounds^24–26^. Thus, we speculated that some allelochemicals play an important role in allelopathy of *M. micrantha* and *C. chinensis*.

To answer the above questions, *M. micrantha* and *C. chinensis* were used as research objects, *Chrysanthemum coronarium* was chosen as receiver species to measure the different effects of leaves extracts from two weeds. Then the compounds of extracts identified by gas phase and mass spectrometry (GC–MS) respectively. To investigate the critical compound in the two extracts and ascertain the dominant role in the allelopathic effect of two weeds, two components tested in the above identification procedure were carried out the next physiological and biochemical experiments on *C. coronarium.*

## Methods and materials

### Plant materials

The aerial parts of *M. micrantha* and *I. cairica* were gathered from Hainan University (Hainan province, China, 20° 3.75′ N, 110° 20.13′ E), and the leaves were cut from the vine. Then, the voucher specimen of *M. micrantha* and *I. cairica* were deposited in the Environmental Science and Engineering Laboratory of Hainan University. The numbers of the specimen were MM-20170521-001 and IC-20170521-002 respectively. Seeds of *C. coronarium* were purchased from Wuchang Shenniu Seeds Company (Hubei province, China). Uniform healthy seeds were collected and stored in the refrigerator at 4 °C.

### Preparation of plant extracts

One hundred grams of leaves of *M. micrantha* and *I. cairica* were picked and washed with tap water, then with distilled water and dried for 2 h. Leaves were then manually chopped and placed at the bottom of an airproof glass bottle (1,000 ml), added to 700 ml distilled water into the jar and shaken well. The mixture was sealed and stored in a growth chamber at 25 °C. Every day the mixture was filtered through a Whatman no.1 filter paper and collected into a new glass bottle (2500 ml) on time, and 700 ml of distilled water was added to the glass bottle of the mixture again. Three days later, all supernatants (2,100 ml) were collected and removed to the SHB-IIIG rotary evaporator (EYEL*A*, Japan). Then vacuum-distilled in a 60 °C water bath by N-1100 vacuum pump (EYEL*A*, Japan) until 10 ml of brown crude resinous was obtained, that is, the concentrated plant extracts were 10 g/ml. Finally, the foliage extracts of *M. micrantha* and *I. cairica* from the above-stated were added to distilled water to diluted, with the concentration were 50 mg/ml, 100 mg/ml, 200 mg/ml, and 400 mg/ml respectively, which were optimized from prior experiments, and then stored refrigerator at 4 °C.

### Phytotoxicity Bioassays of extracts of *Mikania micrantha* and *Ipomoea cairica*

An airproof glass bottle (0.3 L) was used to assess the morphological inhibitions of aqueous extracts. Thirty un-sprouted seeds of *C. coronarium* were selected randomly and put in a glass bottle on two sheets of filter paper moistened with 5 ml distilled water. Then 2 ml extracts of *M. micrantha* and *I. cairica* with different concentrations (50, 100, 200, 400 mg/ml) were added separately per glass bottle whereas the control bottle received 2 ml distilled water. For each species, there were five replicates. The bottles were incubated at 25 °C and 70% relative humidity with a photoperiod 12/12 h day/night. During the seed germination and seedling growth, distilled water was added to keep the filter paper moist. Seed germination was counted daily. When the radicle length was over 2 mm, the seeds were considered as germinated. The germination rate was represented by the number of seeds germinated in a day divided by the number of total seeds (30). Besides, the fresh weight, root and shoot lengths were measured after 7 days. Among them, the fresh weight was obtained by subtracting the weight of filter paper from the weight of plant seedlings + filter paper, and the length of root and shoot were measured with calipers (V5964-A150mm-1EA, Aladdin, China)^27^.

### Preparation of organic extracts

Initially, 300 ml extracts (10 g/ml) of *M. micrantha* and *I. cairica* were filtered through a water-phase filter, then subjected to chromatography column at a flow rate of 150 ml/h for elution after the resin was activated. Then these eluants were re-filtered through 0.45 μm PTFE membrane in a Buchner funnel coupled to a vacuum that collected supernatants. After this, the supernatants were dried in a rotary evaporator until they became solid before re-dissolved by dichloromethane in a test tube (15 mm × 150 mm). Afterward, the mixture was consecutively dissolved in acetonitrile (500 μL) and derivatizing reagent (99% BSTFA + 1% TMCS, 500 μL) for 1 h at 70 °C. At the end of the process, the solutions were re-filtered through 0.45 μm PTFE membrane and saved in an airproof glass bottle after cooled, and then stored refrigerator at 4 °C^28,29^.

### The GC–MS analysis of organic extracts

Samples of the extracts of *M. micrantha* and *I. cairica* 1 μL were injected into a 7890A gas chromatograph equipped with a splitless injector coupled with a 5975C mass spectrometer (Agilent, USA). The mass spectrometer was operated in the electron ionization (EI) mode (70 eV). Helium (99.999%) was employed as a carrier gas, and its flow rate was adjusted to 1 ml/min. The chromatographic separation was performed on an HP-5 MS column (30 m × 250 μm × 0.25 μm, Agilent). The initial temperature of the column was set at 80 °C and held for 3 min, then increased by 8 °C/min to 160 °C and maintained for 2 min, followed by a rise to 240 °C at a rate of 5 °C/min. The temperatures of the GC–MS interface, ion source and quadrupole were set to 250, 230, and 150 °C, respectively. The control samples were analyzed under the same conditions. The total ion flow chart was automatically integrated into Chemstation software (Agilent, USA). Three methods were used to identify the compounds: searching them in the NIST 2011 standard spectrum library, calculating their Kovats index values^30,31^, and comparing their retention times with those of authenticated standards (Sigma-Aldrich, 95–99%, USA). The relative contents of the substances were calculated by the GC peak area. The external calibration curves were also used to quantify the test concentrations of compounds extracted from the samples.

### Physiological and morphological analyses of the identified compounds from extracts

According to the results of the “Identification of Compounds from The Two Extracts”, benzoic acid and cinnamic acid (Sigma-Aldrich, 95–99%) were picked for further bioassay experiments, which accounted for the highest relative contents of compounds in *M. micrantha* and *I. cairica* respectively.

Seeds of *C. coronarium* were used to access the morphological inhibitory effects of the extracted compounds. Thirty un-sprouted seeds of *C. coronarium* were selected randomly and then sown in an airtight glass bottle (0.3 L) on two sheets of filter paper moistened with 5 ml distilled water. The treatment concentrations of benzoic acid and cinnamic acid were 10, 50, 100 and 200 mg/ml, which were optimized from prior experiments. Then 2 ml of two compounds of different concentrations were added separately to the glass bottle whereas control bottles received 2 ml distilled water. The culture conditions were the same as the above phytotoxicity bioassay experiment, and the experiment had three replications. One week later, samples were collected to determine the germination and morphological indices ( shoot and root length)^27^.

After 15 days, samples were carried to determine the physiological indices. The malondialdehyde (MDA) content was measured using the thiobarbituric acid (TBA) reaction^32^. Soluble protein was determined by the Coomassie brilliant blue method^33^. Chlorophyll content was determined by spectrophotometrically, according to Amon^34^. Superoxide dismutase (SOD) activity was determined by WST-1 methods (Nanjing Jiancheng Bioengineering Institute, China)^35^.

### Statistical analysis

The allelopathic index (RI) was used to quantify the intensity of the allelopathic effect, which described by the Eq. (1)^36^.

1 $${\text{RI}} = {1} - {\text{C}}/{\text{T}}$$

where C as the control data and T denoted the treatment data. RI > 0 indicated that the germination or seedling of plants was promoted at present, while RI < 0 stated the inhibition effect. The absolute value of RI represented the allelopathic intensity. The volume of sensitive effect (SE) was the sum of the corresponding RI of germination, root, and shoot lengths.

All data were presented as mean ± standard error (se) and tested by IBM SPSS Statistics 20. Significant differences (*p* < 0.05) were analyzed using one-way ANOVA, followed by LSD’s multiple range tests. SigmaPlot 12.5 was used to draw the graph.

## Results

### Effects of extracts on seed germination and seedling growth

Variations in the morphological data revealed the significant impact of extracts from two weeds on the growth of tested seeds. It can be easily seen from Table 1 that both *M. micrantha* and *I. cairica* extracts significantly inhibited the seed germination of *C. coronarium*, especially when the extract concentration was 400 mg/l (*p* < 0.05), the RI of germination rate was − 0.51 and − 0.86 respectively. Similarly, some significantly antagonistic responses were observed in the root and shoot length of *C. coronarium* in the treatment of the two extracts, and the antagonistic effect was enhanced with the concentration of the extracts. Besides, the root length of *C. coronarium* in the soaking of *I. cairica* extracts was always inhibited stronger than the shoot length, and the extract even slightly promoted the growth of the shoot at the concentration was 50 mg/l. In contrast, the inhibition of root length treated by the extract was stronger than the stem length was observed only when the concentration of *M. micrantha* extracts exceeded 200 mg/l. According to the results of SE presented in Table 1, all of them were negative. For the extract concentration of 50 and 100 mg/l, the comprehensive inhibition of the recipient plants by *M. micrantha* was significantly stronger than that of the *I. cairica* (*p* < 0.05). However, when the concentration of the extract was 400 mg/l, the opposite phenomenon was observed.

**Table 1:** Allelopathic effects of extracts from *Mikania micrantha* and *Ipomoea cairica* on seed germination and seedling growth of *Chrysanthemum coronarium.*

Plants	Concentration (mg/ml)	Response index (RI)			Synthesis effect (SE)
		Germination rate	Root length	Shoot length	
*Mikania micrantha*	50	− 0.14 a	− 0.62 a*	− 1.46 a	− 2.12 a
	100	− 0.38 b	− 2.74 ab	− 2.98 ab	− 6.10 b
	200	− 0.42 b	− 4.38 b	− 2.81 a	− 7.60 b
	400	− 0.53 b	− 7.29 c	− 5.09 b	− − 12.91 c*
*Ipomoea cairica*	50	− 0.11 a	− 1.10 a	0.06 a	− 1.16 a*
	100	− 0.11 a*	− 2.00 b*	− − 0.08 a*	− 2.20 a*
	200	− 0.37 a	− 3.97 ab	− 0.81 a*	− 5.18 b
	400	− 0.86 b	− 13.80 c*	− 3.36 b*	− 18.06 c

Lowercase letters represent that different concentrations of plant extracts have a significant difference at *p* < 0.05, and “*” indicates that two extracts have a significant difference at *p* < 0.05.

### Analysis of constituents of extracts

The GC–MS data showed the extracts of *M. micrantha* and *I. cairica* contained a large number of compounds (Table 2). Nineteen compounds from the *M. micrantha* extracts were identified, and more than half of them were acids. The relative contents of benzoic acid and lactic acid were in the top two among them, reaching 19.36% and 15.95%. On the other hand, twenty-three compounds from the *I. cairica* extracts were identified. Similarly, acids were the dominant source in this mixture, the relative content of which was more than 60%. And the average relative content of cinnamic acid ranked the first among the mixture, reaching 47.56%, which were roughly triple as large as the content of 2-methyl phenyl benzoate (15.75%). Therefore, the average concentrations of benzoic acid and cinnamic acid had the maximum value gaps between two weed extracts, which were selected for further experiments.

**Table 2:** Comparison among the compounds from extracts of *Mikania Micrantha* and *Ipomoea cairica.*

Compounds	*Mikania Micrantha*				Compounds	*Ipomoea cairica*			
	RI	RI^lit^	Identification methods	Relative content of compositions (%)		RI	RI^lit^	Identification methods	Relative content of compositions (%)
**Phenols**					**Esters**				
Phenol	1,053	1051^51^	MS/RI/Std	13.45	Methyl benzoate	1,105	1101^52^	MS/RI	0.08
o-Cresol	1,066	1063^53^	MS/RI	8.68	2-Methyl phenyl benzoate	1900	–	MS	15.75
p-Cresol	1,081	1080^53^	MS/RI	6.7	2-Methyl-hexadecane	1658	1655^52^	MS/RI	0.85
2,6-Dimethylphenol	1,108	1107^54^	MS/RI	3.72	Dibutyl phthalate	1915	1914^55^	MS/RI	1.81
2-Tert-butyl-6-methylphenol	2056	–	MS	1	**Alcohols**				
**Esters**					Glycerol	1,132	1129^56^	MS/RI	0.18
Methyl benzoate	1,103	1101^52^	MS/RI	0.03	**Alkanes**				
Methyl cinnamate	1,305	1304^57^	MS/RI	1.97	Dodecane	1,199	1200^58^	MS/RI	1.89
**Alcohols**					Tetradecane	1,402	1400^59^	MS/RI	1.66
Ethylene glycol	1,152	–	MS	3.52	Octadecane	1801	1800^60^	MS/RI	1.05
**Acids**					1-Nonadecene	1892	1893^61^	MS/RI	2.01
Hydroxyacetic acid	1,283	–	MS	4.66	Hexadecane	1785	1784^62^	MS/RI	0.79
Lactic acid	1,490	1,491.1^63^	MS/RI	15.95	**Acids**				
Nonanoic acid	1587	1584.4^63^	MS/RI	0.65	Lactic acid	1,495	1,491.1^63^	MS/RI	0.18
Benzoic acid	1688	1687^64^	MS/RI/Std	19.36	Glycolic acid	1,510	1508.6^63^	MS/RI	0.47
Succinic acid	1759	1757.6^63^	MS/RI	9.88	Decanoic acid	1553	1555^65^	MS/RI	5.6
Lauric acid	1883	1884^63^	MS/RI	1.66	Benzoic acid	1688	1687^64^	MS/RI/Std	0.23
Salicylic acid	1961	1965.3^63^	MS/RI	3.32	Succinic acid	1755	1757.6^63^	MS/RI	2.12
Phthalic acid	2,135	2,137.2^63^	MS/RI	1.69	Cinnamic acid	1787	1786.9^63^	MS/RI/Std	47.56
Gallic acid	2,821	2,822.8^63^	MS/RI	1.03	Lauric acid	1885	1884^63^	MS/RI	5.16
**Alkanes**					Hexadecanoic acid	1937	1938^66^	MS/RI	5.5
Hexadecane	1787	1784^62^	MS/RI	1.9	Stearic acid	2,489	2,489.1^63^	MS/RI	0.69
**Oximes**					Tetradecanoic acid	2,578	2576^67^	MS/RI	0.37
2-Acetylthiophene-O-methyloxime	1975	–	MS	0.84	Tannic acid	3,006	–	MS	1.21
					**Phenols**				
					2,4-di(1,1-dimethylethyl)-phenol	1889	–	MS/RI	0.44
					**Ketones**				
					(4-Hydroxyphenyl)-2-pentan-2-ketone	2,861	–	MS	0.42

*RI* linear retention indices on the HP-5MS column (relative to n-alkanes), *RI*^*lit*^ retention index value was consistent with that in the literature, *MS* by comparing the MS with that in the NIST library, *Std* by comparing time and MS with those of available authentic standards. “–” not found.

### Morphological indices of the compounds from extracts on seedlings

**Figure 1:**
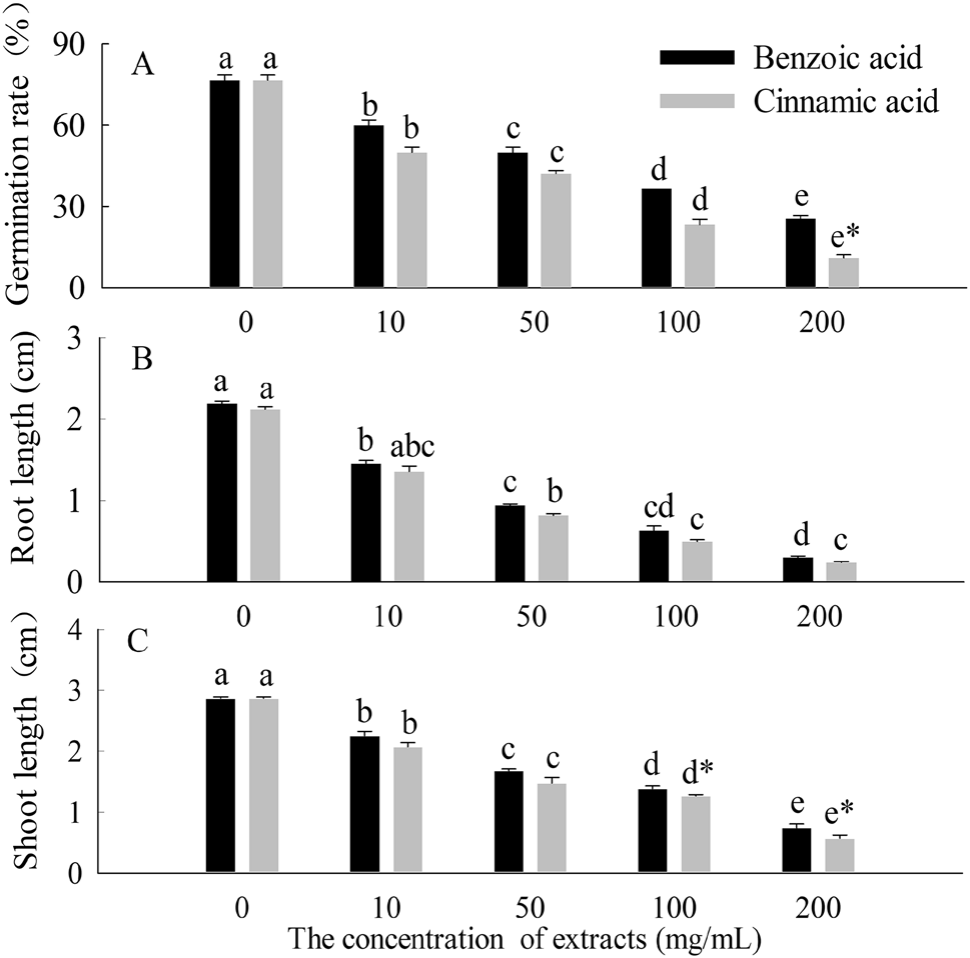
Effects of benzoic acid and cinnamic acid on germination rate (**A**), root length (**B**) and shoot length (**C**) of *Chrysanthemum coronarium* seeds and seedlings. Different lower letters indicate significant differences between the different concentrations of the same allelochemicals at the 0.05 level. The symbol of “*” indicates significant differences between the different chemicals of the same concentrations at the 0.05 level. The same as below.

Concerning the experiments on allelopathic effects of the two invasive plant extracts, the interaction between allelochemicals and concentrations was signification in the parameters evaluated (Fig. 1). The compounds from the extracts were vital inhibited seeds germination under simulating foliage extracts process (*p* < 0.05), which caused the germination decreased by 51.11% and 65.56% respectively at 200 mg/ml compared with control groups in 7 days. At the same time, the root length and shoot length of *C. coronarium* exposed to benzoic acid and cinnamic acid were also substantially suppressed compared to controls, and the inhibited effects were linear to the increasing concentrations of the compounds. In general, cinnamic acid was more inhibitory than benzoic acid (*p* < 0.05).

### Physiological indices of the compounds from extracts on seedlings

**Figure 2:**
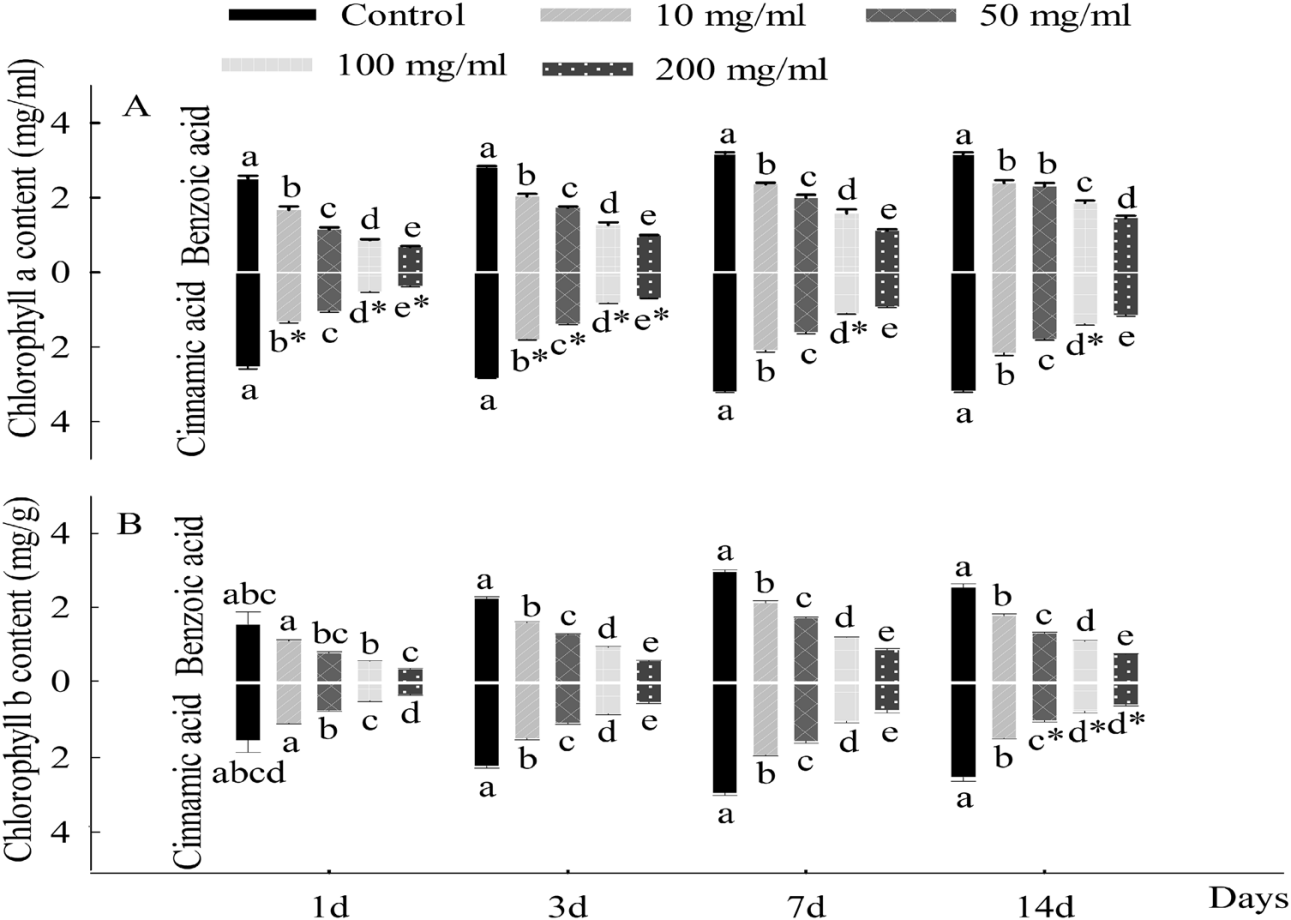
Effects of benzoic acid and cinnamic acid on chlorophyll a (**A**), chlorophyll b (**B**) of *Chrysanthemum coronarium*. Different lower letters indicate significant differences between the different concentrations of the same allelochemicals at the 0.05 level. The symbol of “*” indicates significant differences between the different chemicals of the same concentrations at the 0.05 level. The same as below.

**Figure 3:**
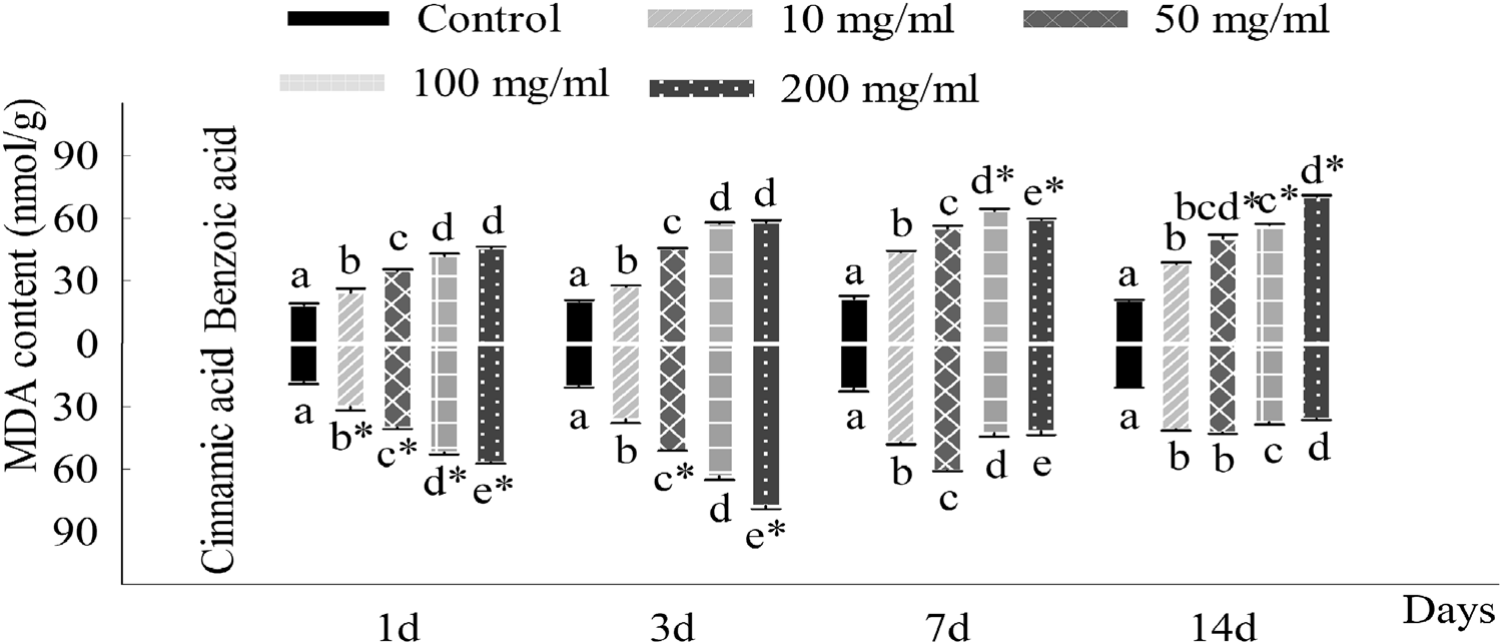
Effects of benzoic acid and cinnamic acid on malonaldehyde (MDA) content of *Chrysanthemum coronarium*. Different lower letters indicate significant differences between the different concentrations of the same allelochemicals at the 0.05 level. The symbol of “*” indicates significant differences between the different chemicals of the same concentrations at the 0.05 level. The same as below.

**Figure 4:**
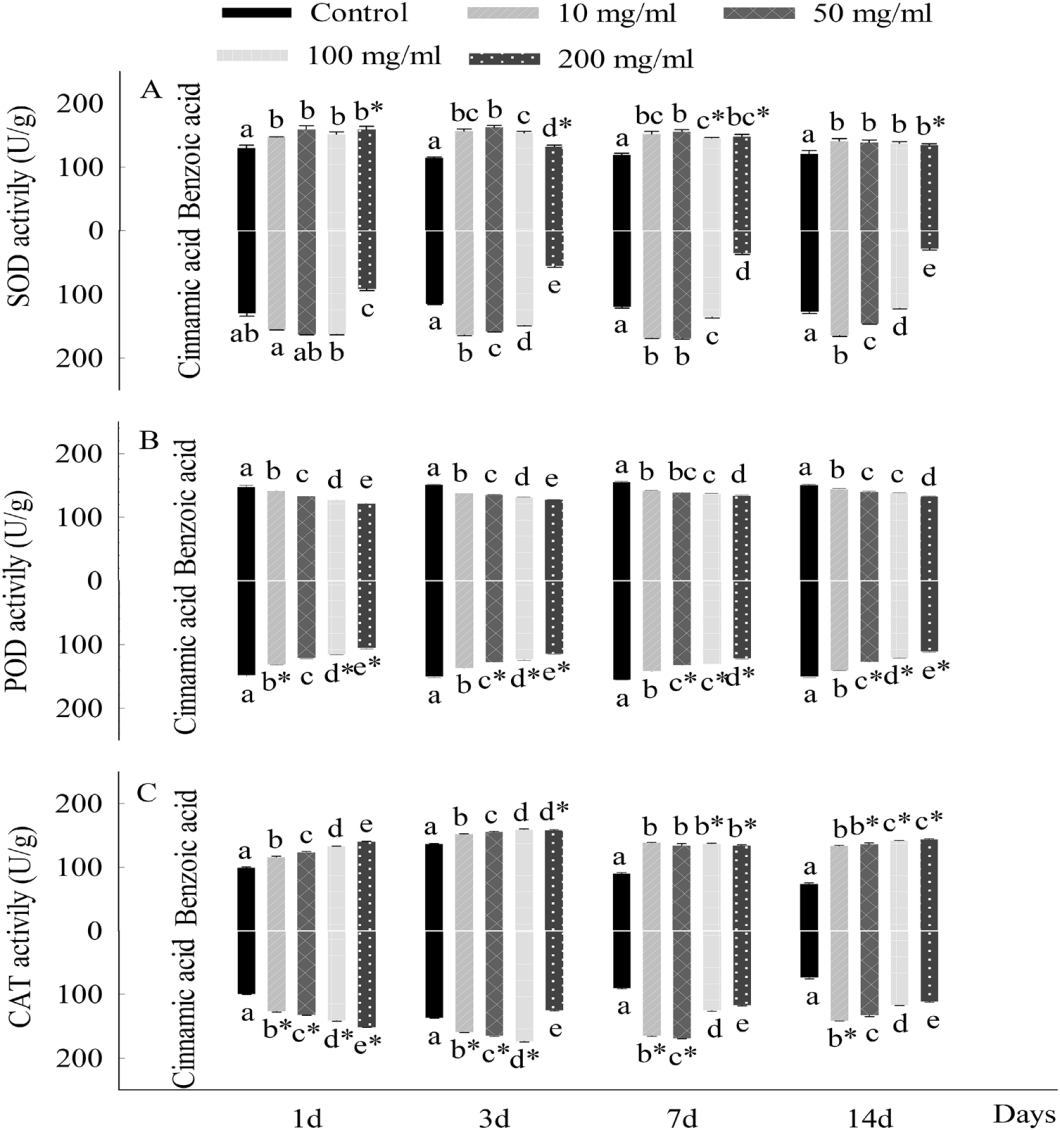
Effects of benzoic acid and cinnamic acid on SOD activity (**A**), POD activity (**B**) and CAT activity (**C**) of *Chrysanthemum coronarium* seedlings. Different lower letters indicate significant differences between the different concentrations of the same allelochemicals at the 0.05 level. The symbol of “*” indicates significant differences between the different chemicals of the same concentrations at the 0.05 level. The same as below.

During the period of seedling growth, there existed substantial differences in the data of parameters evaluated of *C. coronarium* seedlings treated with different concentrations of allelochemicals. The first difference in content was at the content of chlorophyll, in which the content of chlorophyll *a* and chlorophyll *b* was positively linear to the increasing days, but negatively correlated with the concentration of the cinnamic acid and benzoic acid (Fig. 2). At the same time, the inhibited effect of cinnamic acid on chlorophyll a was stronger than that of benzoic acid during seedlings growth, but for chlorophyll b, the significant inhibition of cinnamic acid was stronger than that of benzoic acid appeared at different (50, 100 and 200 mg/l) concentrations in 14th days. Different from the previous parameter, the MDA contents of seedling in the early period (1st and 3rd day) were significantly rose (*p* < 0.05) with the compound concentrations increasing, compared with the control groups (Fig. 3). By contrast, on the 7th and 14th days, the MDA content of *C. coronarium* experienced with 100 mg/l benzoic acid was lower than that with 200 mg/l. Figure 4A, C represented the activity of SOD and CAT, which considerably increased (*p* < 0.05) with the benzoic acid compared to the control groups. However, the contents of SOD of treated groups were all considerably lower than contrast (*p* < 0.05) when seedlings exposed to cinnamic acid at 200 mg/l. Besides, the activity of POD of *C. coronarium* seedlings significantly (*p* < 0.05) declined with the chemical concentration increased, and the inhibited effect of cinnamic acid on chlorophyll a was stronger than that of benzoic acid (Fig. 4B).

## Discussions

Allelopathy is ubiquitous in nature, and most alien plants can make their survival a dominant position through potential allelopathic effects^37^. Our results showed that the extracts of *M. micrantha* and *I. cairica* significantly inhibited the seeds germination and seedling growth of *C. coronarium*, and the inhibitory effects increased with the concentration of extracts. This is consistent with the results of the inhibition of *Brassica rapa* seedling growth by the *I. cairica* and *M. micrantha* aqueous extracts reported in the previous studies^38^. Besides, at the concentration of 50, 100, and 200 mg/ml, the allelopathic effect of *M. micrantha* extracts was stronger than that of *I. cairica*, whereas the inhibition was reversed when concentration was increased to 400 mg/ml. Some studies pointed out that the metabolic system in plants would be disordered when the concentration of foliage extracts reaches a certain threshold^39^. Therefore, we infer the above different experimental phenomenon is related to the tolerance mechanism of plants, and *C. coronarium* is more sensitive to high concentrations of *I. cairica*.

According to the bioassay results of benzoic acid and cinnamic acid extracted from the extracts of *M. micrantha* and *I. cairica* respectively, we found that both allelochemicals significantly inhibited the seeds germination rate and the seedlings morphological indexes of *C. coronarium*. Similarly, as the concentration of allelochemicals increases, the allelopathic effect gradually increases. It was reported that benzoic acid and cinnamic acid were the most main forms of phenolic acids, and they are also important allelochemicals^40^. They can be extracted from the aerial parts of plants and released into the external environment, thus affecting the morphological indicators of crops above and below the ground^41,42^. Phenolic acids can affect seed germination by inhibiting key enzymes required for seed germination, such as phosphorylase^43^. It can also lead to the lack of necessary energy or inhibit the synthesis of some key enzyme intermediates during the germination process, to show a decrease in vigor, germination rate, radicle length and radicle dry weight on crop seeds^44^. We also found that the root growth of *C. coronarium* seedlings was more sensitive to phenolic acids than germination rate and stem length. It was speculated that both benzoic acid and cinnamic acid can significantly inhibit the vertical growth and cell division of the root system, to show significant changes in the root morphology, and thus affect the plant’s absorption of nutrients.

The physiological indexes of crop seedlings are affected by phenolic acid stress. The chlorophyll content of *C. coronarium* decreased with the increase of benzoic acid and cinnamic acid concentration, and the allelopathic effect of cinnamic acid was significantly stronger than that of benzoic acid, which was basically in agreement with Fu et al.^45^. Besides, in terms of seedling growth stage, the chlorophyll content of leaves showed a trend of first increasing and then decreasing, reaching the peak value on the seventh day, which was attributed to a high concentration of allelochemicals and prolonged exposure to allelochemicals causing an impaired chlorophyll synthesis, decreased stomatal opening and the stomatal opening ratio in leaves^45^. Besides, allelopathic stress can cause an increase in reactive oxygen species (ROS) in plants, leading to oxidative stress^46^. Thereby plants always enhance stress resistance by regulating antioxidant enzyme activity. In general, our study showed that SOD and CAT activities in leaves of *C. coronarium* were significantly higher than those in the control group during the growth under benzoic acid exposed, and only high concentration (400 mg/l) of cinnamic acid significantly inhibited SOD activity in the leaves, which is similar with Song et al.^47^. When the concentration of allelochemicals is high enough, the internal defense mechanism of plants collapses after reaching a threshold, and its antioxidant enzyme activity drops sharply. In addition, we observed that the POD activity of *C. coronarium* seedlings decreased with the increase of benzoic acid and cinnamic acid concentration, indicating that the POD activity of *C. coronarium* leaves was most sensitive to phenolic acids, followed by SOD activity.

Allelochemicals may destroy the balance between free radical production and scavenging system in plant tissue, thus leading to membrane lipid peroxidation. And malondialdehyde (MDA) is one of its products. The results showed that MDA content in *C. coronarium* leaves increased significantly during benzoic acid and cinnamic acid exposed compared with the control. In the early growth stage (1–3 days), MDA content exposed to cinnamic acid was significantly higher than that of benzoic acid and peaked on the seventh day and the third day respectively. It indicated that phenolic acids could accelerate the peroxidation of membrane lipid in chrysanthemum leaves after the tolerance threshold was reached, the plants could not resist stress through their regulation, and then MDA content began to decrease^48^. Therefore, we concluded that both benzoic acid and cinnamic acid could improve the antioxidant enzyme activity of *C. coronarium* leaves, accelerate the accumulation of reactive oxygen species in cells, and ultimately lead to the destruction of membrane structure and physiological integrity and the increase of membrane permeability.

In terms of the bioassay method used in this experiment, which has been widely used in exploring the allelopathic effect, and has the advantages of simplicity, short period, and high sensitivity^25,27,49^. Whereas the growing conditions of the pot experiment may be more similar to those in the natural environment than that of laboratory^50^. Therefore, the growth of receptor plants under allelopathic stress in the wild environment needs to be further studied. On the other hand, the germination rate was selected as the sensitivity index to evaluate plant allelopathy, whose changes would affect the abundance and competitiveness of recipient plants in the community. SOD CAT, POD, and other biological indicators directly reflect the degree of stress in seeds. However, at present, we have not studied the effect of *M. micrantha* and *I. cairica* extracts on *C. coronarium* seedlings at the molecular level, so the effects on cell micro-ultrastructure, cell division and elongation, growth regulation system and respiration can not be determined. These aspects will be our next research content.

## Conclusions

The two typical invasive weeds in Hainan island, *M. micrantha* and *I. cairica*, their leaves extracts have negative effects on the growth of *C. coronarium* seeds and seedlings, and the allelopathic effect of *M. micrantha* extracts at low concentration was stronger than that of *I. cairica* extracts, while the high concentration showed an opposite effect. According to GC–MS data of leaves extracts from the two leaves, 19 and 23 chemicals were identified respectively, and each represented by benzoic acid and cinnamic acid. These two allelochemicals can lead to a sharp increase in the activity of the antioxidant enzyme, SOD and CAT and the content of MDA in the *C. coronarium*, thus affecting the germination rate of *C. coronarium* seeds and the growth of seedling roots and shoots. What’s more, the inhibitory effect of benzoic acid is stronger than that of cinnamic acid, which is one of the reasons that the allelopathic effect of *I. cairica* is stronger than that of *M. micrantha*.

## Data Availability

The datasets generated during and/or analysed during the current study are not publicly available due to the data also part of an ongoing study but are available from the corresponding author on reasonable request.
